# Observations on the Petechiæ Sine Febre

**Published:** 1803-11-01

**Authors:** A. Edlin

**Affiliations:** Uxbridge


### Observations on the Petechiæ sine Febre, *by Mr.* A. Edlin, *of Uxbridge.*

THIS disease has not been noticed by Physicians till within these few years past, and a particular description of it has not yet been received into any of the works of systematic writers. Such accounts as have been published, were either suggested in the form of notes, or detailed in books, which do not usually fall into the hands of young practitioners.

The first person who noticed and described it was Dr. Graff of Gottingen; and as the absence of fever is a most striking peculiarity in this disease, he gave it the name it now bears. An account of a similar disorder is recorded in the Transactions of the Medical Society of Copenhagen; and Dr. Tattersall, in the tenth volume of his Medical Commentaries, has described four cases of this complaint, which all terminated in death; but the most accurate and comprehensive account I have seen of the petechiæ sine febre, is given by Dr. Ferris, in the first volume of the Medical Facts and Observations, and the plan he recommends for the mode of treatment is not only the most judicious that can be devised, but if properly followed up, appears the most likely to remove a disease which, if left to Nature, will most commonly prove fatal.

It usually begins with a sense of lassitude and disinclination to active exertion, but the appetite, notwithstanding, continues good, the pulse natural; neither is any pain, fever, or disturbance in the constitution excited. In general, the first untoward symptom is a few bright red spots on the feet and legs, which are scarcely noticed. As the disease advances, they increase in number, put on a purple appearance, and sometimes spread over the whole surface of the body, in which state they have been known to remain for days or even weeks, the patient pursuing his ordinary occupations, insensible of his danger. The eruption now becomes of a livid hue, and sometimes a number of these spots run into one another, and occasion on the lower extremities a dark coloured appearance similar to a bruise; this shall remain in an indolent state for some time, when a thin bloody serum is discharged; on the skin being removed, an ill conditioned sore is observed underneath, which soon becomes exquisitely painful, spreads unequally, and assumes every appearance of a phagedenic ulcer, which seldom heals till the breaches in the constitution are repaired.

repaired. At other times the throat is particularly affected, the tonsils swelling considerably, and assuming a livid aspect; and if the tumefaction does not speedily subside, the cutaneous vessels rupture, and an ill conditioned ulcer appears upon the surface, which occasions the breath to be excessively offensive; or if ulceration does not ensue, an hæmorrhage not unfrequently takes place from these parts. Faintness and languor now succeed, which are followed by loss of appetite and great prostation of strength. An hæmorrhage from the nose now usually follows, which excites a considerable degree of alarm from the difficulty of restraining it. Again and again it takes place, most frequently in the sleep, and is restrained when the patient is in an erect posture; the hæmorrhage continuing, the debility increases, the spots assume a dusky brown colour, bordering upon black, the extremities become cold, and death in a short space of time closes the scene. Such is the usual progress of this disease when left to Nature; but when the complaint takes a favourable turn, the petechial spots diminish in size and number, the bruised appearances upon the legs gradually disappear, or if they have ulcerated begin to heal; the throat no longer continues sore or swelled, the symptoms of debility decline, and that peculiar languor, which characterises diseases of debility, no longer manifests itself.

The petechiæ sine febre very frequently * occurs among the wretched poor in large towns, particularly in London; and the persons most liable to its attacks, have been either previously debilitated by disease, or lived in confined situations, upon a sparing unwholesome diet, by which means their blood is brought into such a thin dissolved state, that it stagnates † in the cellular membrane, and produces petechiæ from a torpor or paralysis of the absorbent mouths of the veins. Such being the case, it must be the business of the physician to prescribe such remedies as will tend to support the patient's strength, and restore to a healthy state the absorbent system. In general it is the most advisable to begin with a gentle emetic, and afterwards to evacuate the contents of the bowels with a lenient cathartic; then the bark, both in decoction and powder, joined with muriatic acid, must be given in as large a quantity as the stomach will bear. If it should occasion a diarrhœa, a few drops

* Critical Review. N. A. Vol. 17. p. 260.

† Darwin's Zoonomia, Vol. 2. Cl. 1. 2. 1. 5.

drops of the tinct. opii. should be added to each dose, according to the age and constitution of the patient; and where the symptoms of debility are very great, moderate doses of opium will be found particularly serviceable. The diet should be nourishing and of easy digestion, and malt liquors, port wine, artificial Seltzer water, oranges, and other ascescent fruits may be taken almost ad libitum. When the throat is affected, the best gargle is a strong decoction of roses acidulated with the muriatic acid, and the sores should be dressed with straps of adhesive plaster renewed every second day, as they tend to check the discharge, and promote cicatrization. By a perseverance in these remedies, the patient's strength and spirits will be found to improve daily, and in a few weeks, with the assistance of fresh air, exercise, and cheerful society, the complaint will be entirely subdued. The following cases will serve to illustrate the foregoing observations; and the last one in particular, exhibits a melancholy proof of the fatal effects of this disease when left to the efforts of Nature alone.

Case I. Miss. H. H. aged 15, of a thin delicate habit of body, perceived about the 10th of last August several small spot upon her ancle, which she did not take particular notice of, supposing them to be flea bites; but as they increased both in size and number every day until the 15th, I was requested to examine them, and observed that the whole of her legs, from the ancle to the knee, were covered with petechial spots, some of them red, others purple, and a few had a brownish tinge; her general health was tolerably good, appetite unimpaired, tongue moist and clean, and except this appearance she had no marks of disease. An emetic was exhibited in the first instance, and afterwards a gentle laxative. The next day she took a strong decoction of bark joined with the muriatic acid, and I recommended her to take a glass of port wine twice a day. She pursued this plan for a week or ten days, when the spots disappeared and she regained her usual serenity.

On the 14th of September following, I was desired to visit her again, the spots having made their appearance a second time in greater numbers, and one upon her ancle of the size of a shilling had ulcerated, and now discharged a thick dark coloured ichor, which corroded the neighbouring skin; the edges were very ragged, and had the appearance of an ill conditioned ulcer; her legs swelled considerably siderably towards night; her countenance was pale and languid, and she complained of a soreness in the throat. On inspecting the fauces, I observed that the tonsils were much swelled, and had a dark coloured appearance, as if they were distended with coagulated blood, and upon the surface of the left one was a small ulcer similar to that on the ancle; the pulse was natural, her appetite not so good as it had been, and she complained of a soreness when she swallowed her food. The tongue was by no means foul, and the heat of the skin perfectly temperate. The bark and a nourishing diet were again had recourse to; the throat was directed to be gargled with infusion of roses, and the sore was dressed with the ung. cer. and a roller applied over it. This plan was pursued till the 20th, when I was sorry to observe that the eruption had extended to the upper extremities; and the sore still continuing to spread, was this day dressed with straps of adhesive plaster, and ℨj. of the pulv. cinchon. was added to each draught. 21st. Her general health is much improved, the spots diminish in size and number, the wound is now healing rapidly; and in the course of a few days more, I had the satisfaction to see her restored to health.

Case II. I was sent for on the 23d of August, 1800, to visit J. W. æt. 33, who was covered from head to foot with petechial spots; those on the neck, arms, and breast, were of a bright red, and those on the legs and feet of a purple colour; he had no fever, pain, or head ache, but was so extremely debilitated as to be almost incapable of rising up in his bed; he had a weak fluttering pulse, and his stomach was so irritable as to be incapable of taking or retaining any solid food. On enquiry, I found that these spots had been out upon him more or less for some time, and as he supposed they arose for want of physic, he took three ounces of Glauber salts, which reduced his strength in a few days surprizingly. I gave him a draught with the decoct. cinchon. and conf. aromat. every four hours, and allowed him a pint of port wine in the day, with panada, sago, and such light nourishment as his stomach would bear. By a perseverance in this plan, the spots in the course of a fortnight disappeared, but several weeks elapsed before he regained his health and strength.

Case III. T. E. a farmer's boy, æt. 14, was observed for some weeks to go about his work with unusual lassitude; his countenance was pale and languid, and he often bled at at the nose; but as his appetite was good, and he slept as usual, no particular notice was taken of him; however, on the morning of the 14th of April, 1801, he was sent from home with a message, and as he was walking along, his nose began to bleed very profusely, and continued so without intermission till he returned home again; but as an hæmorrhage from the nose is a very common occurrence in young people, little attention was paid to it till he became faint from the loss of blood. He was then put to bed, and while his friends were undressing him, his body was observed to be covered with petechial spots. On being interrogated as to their duration, he replied, that they had been so for several weeks. A friend of mine was then sent for, who saw him in the evening, but the patient was now convulsed, and in the course of a few hours expired. In this case the patient was evidently lost from neglect; had proper medical assistance been called to him when the petechial spots first made their appearance, in all probability the lad's death might have been prevented.

